# Real-Life Self-Control is Predicted by Parietal Activity During Preference Decision Making: A Brain Decoding Analysis

**DOI:** 10.3758/s13415-021-00913-w

**Published:** 2021-06-01

**Authors:** Klaus-Martin Krönke, Holger Mohr, Max Wolff, Anja Kräplin, Michael N. Smolka, Gerhard Bühringer, Hannes Ruge, Thomas Goschke

**Affiliations:** 1 Faculty of Psychology, Technische Universität Dresden, 01062 Dresden, Germany; 2 Department of Psychiatry and Psychotherapy, Technische Universität Dresden, Dresden, Germany; 3 Neuroimaging Center, Technische Universität Dresden, Dresden, Germany; 4 Department of Clinical Research, Faculty of Health, University of Southern Denmark, Odense, Denmark

**Keywords:** Self-control, Value-based decision making, Ecological momentary assessment, Ecological validity, Brain decoding

## Abstract

Despite its relevance for health and education, the neurocognitive mechanism of real-life self-control is largely unknown. While recent research revealed a prominent role of the ventromedial prefrontal cortex in the computation of an integrative value signal, the contribution and relevance of other brain regions for real-life self-control remains unclear. To investigate neural correlates of decisions in line with long-term consequences and to assess the potential of brain decoding methods for the individual prediction of real-life self-control, we combined functional magnetic resonance imaging during preference decision making with ecological momentary assessment of daily self-control in a large community sample (N = 266). Decisions in line with long-term consequences were associated with increased activity in bilateral angular gyrus and precuneus, regions involved in different forms of perspective taking, such as imagining one’s own future and the perspective of others. Applying multivariate pattern analysis to the same clusters revealed that individual patterns of activity predicted the probability of real-life self-control. Brain activations are discussed in relation to episodic future thinking and mentalizing as potential mechanisms mediating real-life self-control.

**Supplementary Information:**

The online version contains supplementary material available at 10.3758/s13415-021-00913-w.

## Introduction

Self-control, the ability to adjust behavior according to long-term goals despite short-term temptations, is associated with a wide range of positive real-life outcomes (De Ridder et al., 2011; Moffitt et al., 2011; Tangney et al., 2004). According to the valuation model of self-control (Berkman et al., 2017), self-controlled choice involves a dynamic integration process wherein subjective value for each choice option is calculated by integrating various short- and long-term gains and costs. The resulting integrated value signal reflects the individual’s preferences at the moment of decision and biases the individual toward the enactment of the most valued option. We thus assume that individual differences in self-control reflect the relative weight with which long-term consequences enter this value integration process (Krönke et al., 2020a). While empirical evidence supports the hypothesis that self-control is finally based on a valuation signal computed in the ventromedial prefrontal cortex (vmPFC), it remains unclear *how* exactly this valuation signal is computed.

According to dual-process theories, self-control often is reduced to the inhibition of impulsive behaviors (Heatherton & Wagner, 2011; Hofmann et al., 2009; Metcalfe & Mischel, 1999). While inhibitory control is certainly one route of self-control, there are multiple alternative strategies of successful self-control (Fujita, 2011), e.g., by anticipation of future outcomes, which is necessary to pursue long-term goals (Goschke, 2014; Krönke et al., 2020b; Kruschwitz et al., 2018; Soutschek et al., 2016). At the neurobiological level, there is large consensus that cognitive control regions, such as the prefrontal cortex (PFC), play a major role in self-control. Previous research revealed that the inferior frontal gyrus is critical for response inhibition (Aron et al., 2014) and that the dorsolateral PFC is involved in active goal-maintenance (Miller & Cohen, 2001). With regard to the valuation model of self-control, it has been suggested that the role of the dorsolateral PFC is the top-down modulation of the value signal in the vmPFC in order to strengthen values assigned to long-term goals (Hare et al., 2009; Hare et al., 2011; Hare et al., 2014). Less is known about the contribution of nonexecutive parietal brain regions to self-control. One possibility includes that parietal brain regions, such as the posterior inferior parietal lobe and precuneus, mediate self-control by the representation of future outcomes (Andrews-Hanna et al., 2010; Benoit & Schacter, 2015; Kruschwitz et al., 2018; Soutschek et al., 2016; Xu et al., 2016; Zwosta et al., 2015; Zwosta et al., 2018). This also could involve interactions with the dorsolateral PFC, for instance a vivid imagination of future consequences might increase the representation of long-term goals in the dorsolateral PFC.

While significant progress has been made in identifying the neural basis of self-control in the laboratory (Turner et al., 2019), the ecological validity of neural correlates of in-laboratory self-control remains largely unknown. However, ecological validity has become increasingly debated since concerns were raised that task-based measures of self-control lack reliability (Eisenberg et al., 2019; Enkavi et al., 2019) and are not meaningfully associated with self-reported self-control (Saunders et al., 2018). So far, only a few studies in self-control research have addressed the trade-off between experimental control and ecological validity by combining neuroimaging methods with the assessment of real-life behavior by using smartphone-based ecological momentary assessment (EMA) (Berkman et al., 2011; Krönke et al., 2018, 2020a, 2020b; Lopez et al., 2014).

Recently, we used a ROI-approach to investigate, whether the weight with which long-term consequences modulate value signals in the vmPFC during decision making is predictive of individual differences in real-life self-control (Krönke et al., 2020a). Here, we go beyond previous work in several ways. First, while we previously investigated the modulation of value signals in the vmPFC and its role in predicting real-life self-control, the present paper focusses on the role of prefrontal and parietal cortex regions, which are involved in inhibitory control and future-directed thinking. Second, going beyond the ROI-based approach in our previous publication, we examined whether it is possible to use brain-decoding methods to predict real-life self-control at the individual level. An exploratory whole-brain analysis was conducted contrasting decisions in line with long-term consequences and decisions in line with short-term consequences, a contrast that has not been analyzed so far. We hypothesized that decisions associated with long-term consequences in the laboratory should be associated either with stronger activation of lateral prefrontal brain regions (supporting the role of inhibitory control for self-control) and/or, alternatively, with activation in the posterior inferior parietal lobe and precuneus (in support of other self-control strategies, such as value modulation via the anticipation of future outcomes).

To predict real-life self-control at the individual level, multivariate pattern analysis (Mwangi et al., 2014) was applied to the functional magnetic resonance imaging (fMRI) data and combined with EMA of real-life self-control. We hypothesized that neural correlates of preference decision making in the laboratory should predict individual differences in real-life self-control.

## Method

### Participants

A total of 338 young adults (aged 19-27 years; 199 females) were recruited from a random community sample from the city of Dresden, Germany, for a multiyear, longitudinal research project on the role of cognitive control and decision making in self-control failures and addictive behaviors (for previous publications from this project, see Kräplin et al., 2020; Krönke et al., 2018, 2020a, 2020b; Wolff et al., 2016; Wolff et al., 2020). Note that the sample size in the present study is larger than in our previous neuroimaging publications (Krönke et al., 2018, 2020a), as in the meantime, data acquisition from all participants enrolled in this project has been completed. Participants were paid 40 Euro for completing the scanning session and EMA. All participants provided written, informed consent. The study was approved by the local research Ethics Committee of the Technische Universität Dresden (EK45022012) and conducted in accordance with the Declaration of Helsinki. Participants were excluded if they had neurological conditions that might affect cognition or motor performance, magnetic-resonance contraindications, lifetime schizophrenia or psychotic symptoms, bipolar disorder, and if they had somatoform, anxiety, obsessive-compulsive or eating disorders, or major depression in the past 4 weeks. For the current fMRI study, all participants who had completed the fMRI session (N = 313) were included. After data collection, 25 participants were excluded due to excessive movement (>3 mm in translation or rotation) or high amount of missed trials (more than 15%, i.e., 18 trials or more than 9 missed trials in a row) during acquisition of fMRI data, and 7 participants were excluded due to floor or ceiling effects (i.e., no trials indicating self-control success/self-control failure). FMRI data were thus analyzed for the remaining 281 participants. Due to incomplete acquisition of EMA data for nine participants and zero reported conflicts for six participants, the final sample for the prediction of real-life self-control was 266 participants (aged 19-27 years; 157 females).

### Assessment of real-life self-control via ecological momentary assessment

**Fig. 1:**
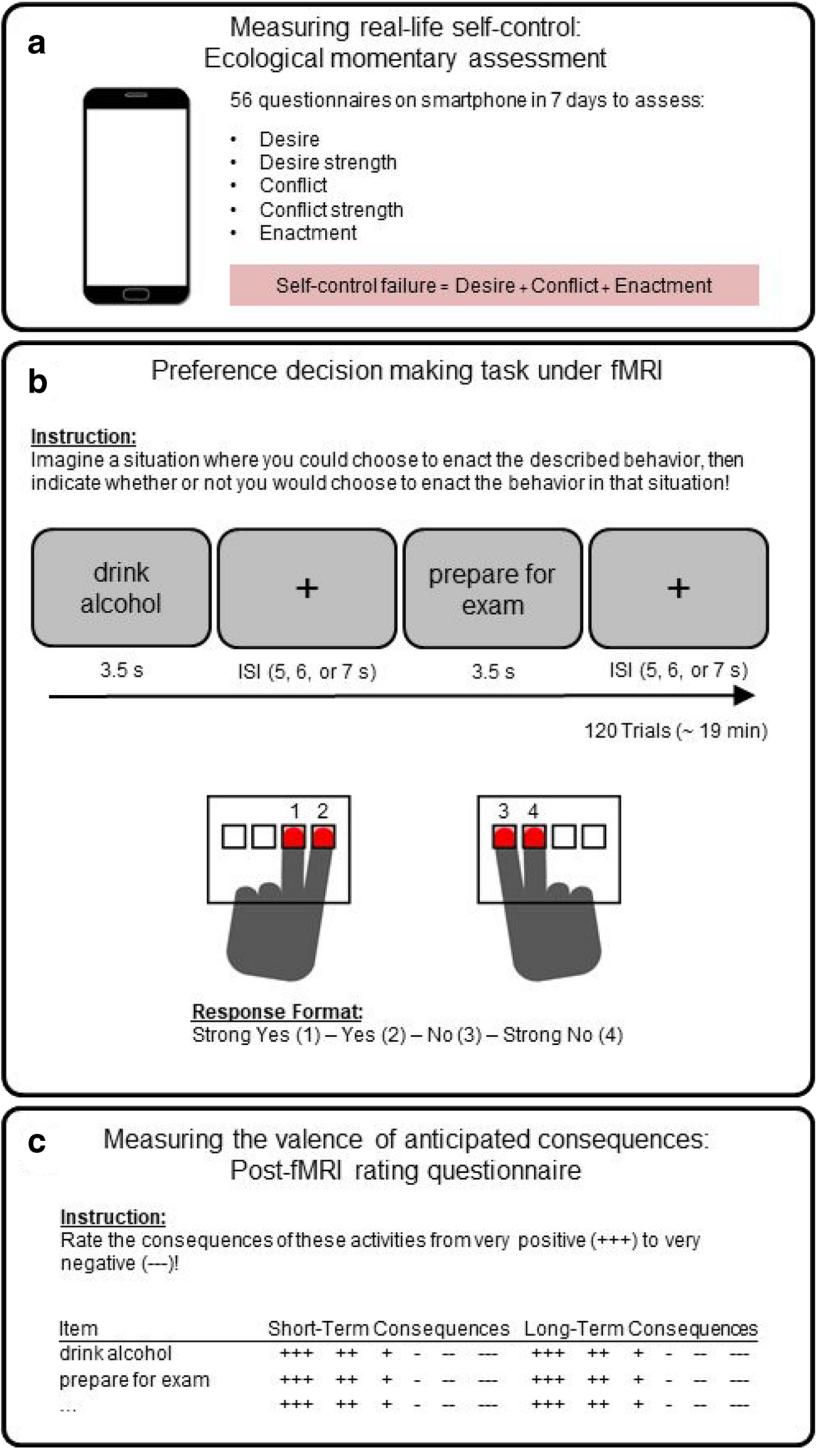
Overview of data collection. **(A)** Ecological momentary assessment was used to acquire data on real-life self-control. **(B)** Preference decision making task during fMRI: Participants were instructed to imagine a realistic situation where they have the option to enact a specific behavior and then to make a decision indicating their preference. **(C)** Measuring the valence of anticipated consequences in a paper and pencil questionnaire: Participants were asked to rate the previously seen items with regard to their anticipated short- and long-term consequences on six-point scales ranging from very positive (+++) to very negative (−−−). By combining decisions and ratings, it was possible to define decisions in line with long-term consequences and decisions in line with short-term consequences

Real-life self-control failures were assessed by using an EMA procedure adapted from (Hofmann et al., 2012) (Figure 1A). Participants were provided smartphones equipped for EMA (using the customizable application movisens XS), which they carried with them continuously for 7 days. Eight alarms per day were issued randomly within a 14-hour time window starting at either 8, 9, or 10 a.m., depending on participants’ habitual waking hours. Upon accepting an alarm, participants were prompted to complete a short questionnaire on the device to examine the occurrence of self-control conflicts in the hour preceding the alarm. First, they were asked whether they had experienced a desire within the past hour. If they reported a desire, they were asked to indicate the strength of the desire on a scale from 1 (very weak) to 6 (very strong), to select the respective type of desire from a list of 19 categories (e.g., eating, sleeping, drinking, smoking, etc.), and to indicate whether the desire was in conflict with a superordinate goal. If they reported a conflict, participants were asked to rate the conflict strength on a six-point scale from 1 (very weak) to 6 (very strong), to indicate whether they had attempted to resist the desire, and whether they had enacted the desired behavior. The questionnaire is summarized in Appendix A (Table A1). Depending on response rates, each participant completed up to 56 questionnaires. Self-control failures were operationalized as occasions where participants enacted conflict-laden desires. For each participant, the probability of a self-control failure in a conflict situation was computed as the relative amount of self-control failures in conflict situations.

### Self-reports of trait self-control

Participants’ self-evaluations of trait self-control were assessed with a German version of the Brief Self-Control Scale (BSCS; Bertrams & Dickhäuser, 2009; Tangney et al., 2004). The BSCS comprises 13 items (e.g., “I am good at resisting temptation”; “People would say that I have iron self-discipline“; “Sometimes I can’t stop myself from doing something, even if I know it is wrong“). High BSCS scores indicate high levels of trait self-control.

### Preference decision-making task

Neural correlates of preference decision making were measured using BOLD-fMRI in a task (Krönke, 2020a) in which participants indicated their subjective preference for a broad range of daily behaviors (Figure 1B). Participants were shown short, verbal descriptions of 40 actions with potentially diverging short- and long-term consequences (e.g., “drink alcohol”; “prepare for exam”; “play videogames”; or “clean kitchen”; for a list including all items see Table B1 in Appendix B). Participants were instructed to imagine for each item a realistic choice situation and then to decide whether they would perform the action and indicate the strength of their preference on a four-point scale (strong yes; yes; no; strong no). To facilitate interpretation of the subsequent parametric analysis of decision value, the scale was recoded, so that higher numbers indicated higher decision value (strong no = −2; no = −1; yes = 1; strong yes = 2). Each trial consisted of the visual presentation of the item (3.5 s) followed by a fixation cross (jittered interstimulus-interval [ISI] of 5 s, 6 s, or 7 s; average ISI = 6 s), yielding an average trial length of 9.5 s. Note that once participants indicated their response, the item remained visible on the screen until the end of the 3.5-s stimulation period. Forty different items were randomized and repeated three times, yielding a total number of 120 trials and a total duration of 19 min.

After fMRI-scanning, participants rated the same 40 items with regard to the value of their anticipated short- and long-term consequences on a six-point scale from “very positive” to “very negative” (Figure 1C); see Appendix C for more details about the rating procedure. The scale was recoded so that higher numbers indicated more positive ratings (very negative = −3, very positive = 3). Ratings were performed after the fMRI task to avoid that the decision process during fMRI was influenced by ratings.

Based on these ratings, for each participant items were individually classified as items with divergent consequences when the participant assigned a positive value to anticipated short-term consequences and a negative value to anticipated long-term consequences, or vice versa. Decisions on trials with divergent consequences were further classified as decisions in line with long-term consequences when the participant declined a decision associated with negative long-term outcomes or accepted a decision associated with positive long-term outcomes.

### fMRI Data Acquisition

Functional images were acquired using a T2*-weighted, gradient-echo, echo planar imaging (EPI) sequence (TE = 25 ms, TR = 2 s, flip angle 78°, slice thickness 3.2 mm, matrix 64 x 64, FOV 19.2 cm, in-plane resolution 3 x 3 mm) on a Siemens MAGNETOM Trio A Tim 3 T scanner with a 32-channel head coil. Thirty-four axial slices, oriented parallel to the AC-PC line covering the whole brain, were acquired. In addition, high-resolution anatomical images were acquired (TE = 2.26 ms, TR = 1,900 ms, flip angle 9°, matrix 256 x 256, FOV 25.6 cm, 591 sagittal slices, slice thickness 1 mm).

### fMRI Data Analysis

*SPM12* (www.fil.ion.ucl.ac.uk/spm/) and Matlab2018b were used for preprocessing and statistical analyses of fMRI data. Each participant’s structural image was co-registered with the functional images. Functional images were slice time corrected, spatially realigned, and unwarped using field maps. Spatial normalization to Montreal Neurological Institute (MNI) space was performed using the unified segmentation approach (Ashburner & Friston, 2005), which is based on the separation of gray matter, white matter, and cerebrospinal fluid (voxel-size 3 mm). Images were spatially smoothed using an 8-mm, full-width, half-maximum (FWHM) Gaussian filter. For baseline correction data were high-pass-filtered with a cutoff period of 128 s.

A general linear model (GLM) of blood oxygen level-dependent activity was computed, including eight task regressors: (i) decisions in line with long-term consequences A (participant declines activity with positive short-term but negative long-term consequences), (ii) decisions in line with long-term consequences B (participant accepts activity with negative short-term but positive long-term consequences), (iii) decisions associated with short-term consequences A (participant accepts activity with positive short-term but negative long-term consequences), (iv) decisions associated with short-term consequences B (participant declines activity with negative short-term but positive long-term consequences), (v) decisions all-positive enactment (participant accepts activity with positive short-term and positive long-term consequences), (vi) decisions all-negative no enactment (participant declines activity with negative short-term and negative long-term consequences), (vii) decisions all-positive no enactment (participant declines activity with positive short-term and positive long-term consequences), (viii) decisions all-negative enactment (participant accepts activity with negative short-term and negative long-term consequences). For subsequent analyses regressors (i) and (ii) were collapsed to indicate decisions in line with long-term consequences; similarly, regressors (iii) and (iv) were collapsed to indicate decisions in line with short-term consequences. The six motion parameters estimated during realignment, session constants, and missed trials were included as regressors of no interest. Moreover, regressors of interest were convolved with a canonical form of the hemodynamic response.

In exploratory whole-brain first-level analyses, decisions associated with long-term consequences were contrasted with decisions associated with short-term consequences for each subject (decisions long-term > decisions short-term). These contrast images were then entered into second-level analyses, and paired *t*-test were used to test for effects between conditions. Clusters were obtained using a whole-brain voxel-threshold of *p* < 0.001 with a minimum cluster size of 270 mm^3^. Family-wise error (FWE) correction based on random field theory was applied at the voxel-level (*p* < 0.05).

### Prediction of real-life self-control failures by multivariate pattern analysis

Clusters obtained during second-level analyses (decisions long-term > decisions short-term) were used as ROIs for multivariate pattern analysis. Please note that by using test set data for feature selection, the resulting coefficients will provide an accurate estimate of the true, error-free effect size; however, this approach may lead to a better performance compared with using genuinely new data for feature selection. For the multivariate pattern analysis, the same images (normalized to MNI space, 8-mm FWHM smoothing) were used as for the univariate analysis. To predict the probability of real-life self-control failures based on individual activity patterns in these ROIs, L1-regularized logistic regression (implemented via the Matlab function *lassoglm*) was used. A leave-one-subject-out cross-validation routine was used, where a single subject’s data were excluded. Then, a model was trained with all of the remaining subjects’ voxel-wise data. The model was applied to the left-out subject (see the Supplemental Material for an alternative, k-fold, cross-validation procedure). This routine was repeated for each subject in the final sample (N = 266), generating cross-validated predictions. To assess the predictive power of the model, predicted real-life self-control failures were correlated with observed self-control failures. The L1-regularization parameter *λ* was optimized via nested leave-one-subject-out cross-validation loops on the respective training data. This computationally expensive parameter selection procedure was implemented to provide unbiased parameter estimates (Kriegeskorte et al., 2009). The *λ* parameter was optimized along the parameter range {2^−8^, 2^−8.5^, 2^−9^, ⋯, 2^−13.5^}. Statistical significance of the correlation between predicted and observed real-life self-control failures was determined by a permutation test. To sample from the null distribution, the predicted real-life self-control failures values were randomly permutated and correlated with the actual real-life self-control failure values 1,000,000 times. The *p*-value was computed as the fraction of correlation values sampled from the null distribution that was larger than or equal to the actual correlation value.

## Results

### Decisions in line with long-term consequences during the preference decision-making task

On average, items involved diverging consequences in 61.83 (*SD* = 22.08, range = 5-110) of 120 total trials, i.e., in 51.53% of trials (*SD* = 18.4). In 27.26 trials (*SD* = 16.17, range = 2-80), i.e., in almost 50% of the trials with diverging consequences, participants indicated that they would make a decision in line with long-term consequences in the imagined situation (enacting a behavior that was rated to have negative short-term consequences, or not enacting a behavior that was rated to have positive short-term consequences). In 34.57 trials, (*SD* = 14.91, range = 3-79), participants indicated that they would make a decision in line with short-term consequences. The average proportion of missed responses was low (*M* = 2.59, *SD* = 3.0). The number of trials with diverging consequences during the task was positively correlated with the number of real-life conflicts investigated using EMA (*r* = 0.168; *p* = 0.006; *N* = 266). The number of decisions in line with short-term consequences during the task was positively correlated with the number of real-life self-control failures, investigated using EMA (*r* = 0.205; *p* = 0.001; *N* = 266). The number of in-task decisions in line with short-term consequences was negatively correlated with trait self-control in terms of BSCS scores (*r* = −0.253; *p* = 0.000; *N* = 280).

### Real-life self-control during ecological momentary assessment

On average, participants responded to 43.97 (*SD* = 8.98) of the 56 issued alarms (78.5%) and reported 31.85 (*SD* = 9.5) desires, 11.61 (*SD* = 7.28) of which were conflict-laden (36.5%). Of the conflict-laden desires, 6.17 (*SD* = 4.82) were enacted (53.1% self-control failures). The number of real-life self-control failures (*r* = −0.156; *p* = 0.011; *N* = 266) was negatively correlated with trait self-control.

### Neural correlates of decisions in line with long-term consequences and the prediction of individual real-life self-control by multivariate pattern analysis

**Fig. 2:**
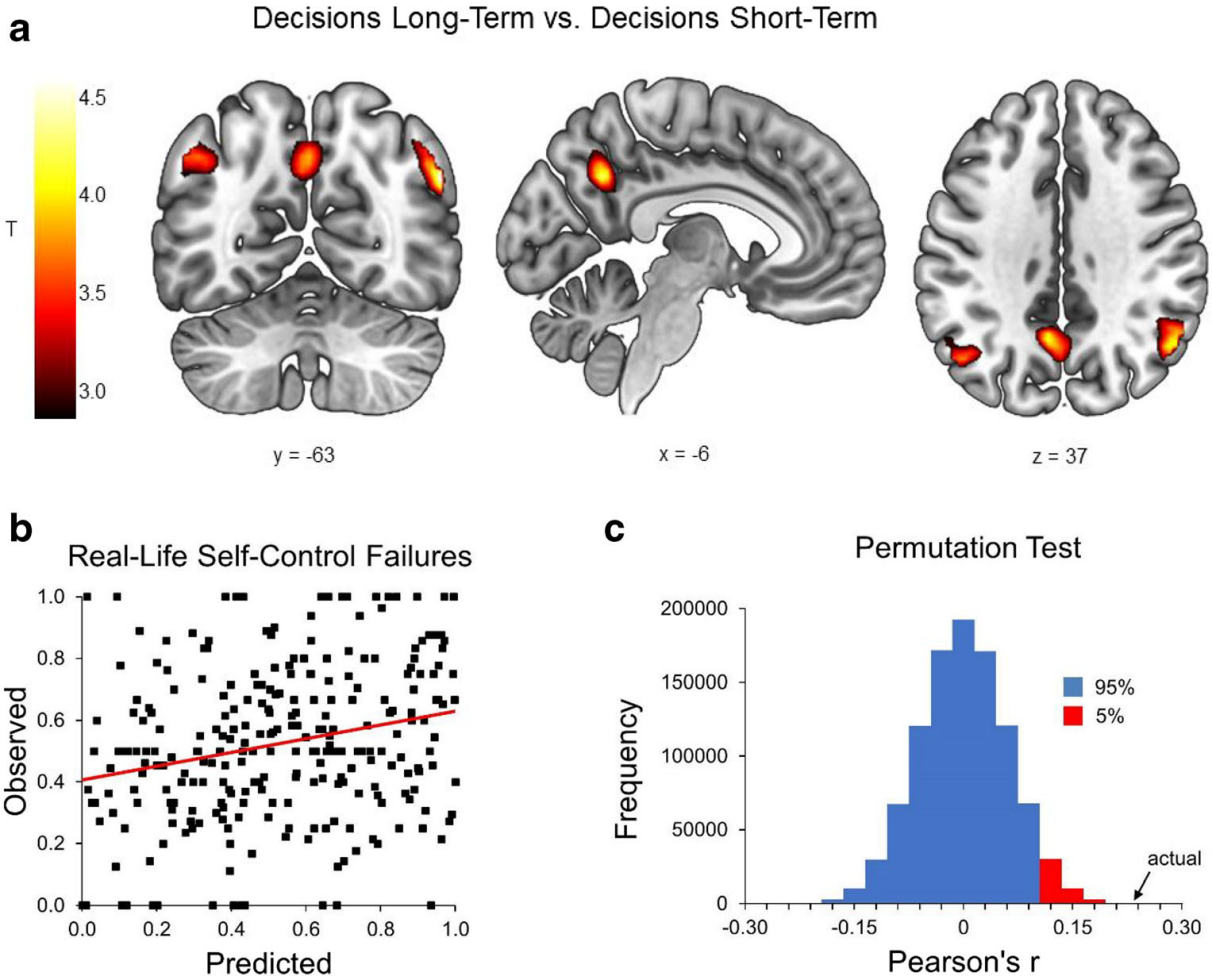
**(A)** In a preference decision-making task (*N* = 281), univariate analyses revealed stronger activation in bilateral angular gyrus and in the precuneus for decisions in line with long-term consequences versus decisions in line with short-term consequences (thresholded at *p* < 0.001 unc.). **(B)** Prediction of real-life self-control by multivariate pattern analysis (*N* = 266) was correlated with observed self-control as measured by ecological momentary assessment (*r* = 0.243, RMSE = 0.32). **(C)** Statistical significance of the prediction was determined by a permutation test. The approximate null distribution shows that the actual correlation, indicated by the black arrow, is significant (*p* < 0.00005)

Contrasting decisions in line with long-term consequences and decisions in line with short-term consequences revealed three clusters where activity was stronger for decisions in line with long-term consequences compared with decisions in line with short-term consequences: bilateral angular gyrus (right hemisphere peak: [54, −61, 32], *t* = 4.71, *p* < 0.05 FWE-corrected at the voxel-level; left hemisphere peak: [−45, −67, 41], *t* = 4.58, *p* < 0.05 FWE-corrected at the voxel-level) and precuneus (peak: [−6, −58, 35], *t* = 4.42, *p* < 0.05 FWE-corrected at the voxel-level) (Figure 2A). Importantly, applying multivariate pattern analysis to those three clusters revealed that individual patterns of activity also predicted the probability of real-life self-control failures significantly above chance (*r* = 0.243, *p* < 0.00005; RMSE = 0.32) (Figures 2B and C; see Figure S1 in the Supplemental Material for consistent results using k-fold cross-validation). See the Supplemental Material for two additional post-hoc MVPA investigating the contribution of the precuneus versus (bilateral) angular gyrus clusters and for the prediction of real-life self-control failures based on univariate analyses.

## Discussion

The present study pursued two goals: (i) to identify neural correlates of decisions in line with long-term consequences in the laboratory; and (ii) to investigate the potential of brain decoding measures for the prediction of real-life self-control at the individual level. There were two main results: First, there was increased activation for decisions in line with long-term consequences in bilateral angular gyrus and precuneus. Second, multivariate pattern analysis revealed that individual activation patterns in the same brain regions predicted individual real-life self-control examined via EMA.

Activity in the precuneus and angular gyrus has been associated with a wide range of cognitive abilities. The precuneus is involved in visuo-spatial imagery, episodic memory retrieval, self-processing operations, such as first-person perspective taking and experience of agency and self-consciousness (for a review see Cavenna & Trimble, 2006). The angular gyrus is involved in social cognition, default mode network functions, semantic processing, word reading and comprehension, number processing, memory retrieval, attention and spatial cognition, and conflict resolution (for a review see Seghier, 2013). Interestingly, both the precuneus and the angular gyrus have been associated with the anticipation of future outcomes (Andrews-Hanna et al., 2010; Benoit & Schacter, 2015; Kruschwitz et al., 2018; Soutschek et al., 2016; Xu et al., 2016;Zwosta et al., 2015 ; Zwosta et al., 2018), a cognitive function that is necessary for episodic future thinking, i.e., the mental simulation of experiences that may occur in one’s personal future (Goschke, 2014; Schacter et al., 2017). Whereas precuneus and angular gyrus have been identified as parts of the core network of episodic future thinking, their specific contributions remain unknown (Benoit & Schacter, 2015). Previous research suggests that the precuneus is involved in self-processing, namely first-person perspective taking (Cavanna & Trimble, 2006; Xu et al., 2016). Moreover, the role of the angular gyrus has been implicated in future-oriented thinking, i.e., the representation of action outcomes and goal-directed action (Zwosta et al., 2015; Zwosta et al., 2018).

We suggest that, although speculative, episodic future thinking could be the mechanism that enables self-control by increasing the impact of long-term consequences during decision making. This view is consistent with recent evidence that the relative weight with which long-term outcomes modulate value signals in the vmPFC predict individual differences in real-life self-control (Krönke et al., 2020a). Further support comes from a transcranial magnetic stimulation study that showed that disturbing the posterior temporoparietal junction (including the angular gyrus) increases delay discounting, suggesting a causal involvement of the temporoparietal junction in implementing future-oriented behavior (Soutschek et al., 2016). Finally, behavioral evidence has shown that episodic future thinking reduces delay discounting, thus biasing dietary decision away from immediate food rewards and toward longer-term health goals related to weight loss discounting (Dassen et al., 2016; O'Neillet al., 2016; Sze et al., 2017). Analogous effects were observed with regard to consumption of alcohol and cigarettes (Snider et al., 2016; Stein et al., 2016).

Note that the angular gyrus and the precuneus are not only involved in taking the perspective of one’s future self (i.e., episodic future thinking) but also in taking the perspective of other people, which is investigated with theory-of-mind or mentalizing tasks (Buckner et al., 2008; Buckner & Carroll, 2007; Mar, 2011; Spreng et al., 2009). In particular, it has been suggested that the angular gyrus, which is a key node of the *social brain* (Frith & Frith, 2010), is involved in reasoning about other people’s minds (Saxe & Kanwisher, 2003; Saxe & Powell, 2006). Thus brain activity observed in the present study may reflect that participants considered how they would be judged by other people if they accepted behaviors of low social desirability, such as “to take drugs,” “to cheat on somebody,” “to gossip about someone,” or alternatively, if they declined behaviors of high social desirability, such as “to save money,” “to study for an exam,” and “to get up early” (see Table B1 in Appendix B for all task items). Because trait self-control and social desirability are substantially correlated (Tangney et al., 2004), it is plausible that participants who considered how they might be judged by others made more self-controlled decisions in the task and in real-life. In summary, the observed activation of angular gyrus and precuneus can be explained by different forms of perspective taking, such as imagining one’s own future and the perspective of others (Buckner & Carroll, 2007; Spreng et al., 2009).

Another brain region that has been associated with episodic future thinking is the medial PFC (Benoit et al., 2011; Peters & Büchel, 2010). Using monetary intertemporal choice paradigms, it has been shown that farsighted decisions were mediated by increased prefrontal-medial-temporal interactions (Peters & Büchel, 2010) and that the medial PFC mediates the impact of episodic future thinking by representing the reward magnitude of envisaged events (Benoit et al., 2011). The fact that no medial-prefrontal brain activations were observed in the present study might be explained by crucial differences in experimental designs. Note that, although the task required participants to make real choices between temptations and long-term goals, these decisions were still hypothetical and were not followed by real consequences. This is in contrast to previous studies that investigated value-based decision making (Hare et al., 2009; Kable & Glimcher, 2007; Peters & Büchel, 2009) and might explain why in the present study, brain activations were observed in regions related to episodic future thinking but not in the dorsolateral PFC and vmPFC. However, using the same task, but focusing on a different contrast, we recently found that vmPFC activity was parametrically associated with decision value and that the modulation of the vmPFC signal by long-term consequences was associated with real-life self-control (Krönke et al, 2020a).

Finally, the present study contributes to the investigation of individual differences in real-life self-control. While previous studies focused on the role of cognitive control and response inhibition in real-life self-control (Berkman et al., 2011; Krönke et al., 2018; Lopez et al., 2014), only recently the contribution of valuation processes were examined. Using the same task as in the present study, Krönke et al. (2020a) showed that real-life self-control was predicted by ventromedial PFC activity encoding the value of anticipated future outcomes. The present study adds to this literature on real-life self-control and is consistent with the idea that episodic future thinking (i.e., imagining one’s own future), mediated by the precuneus and angular gyrus, might be an additional important aspect in the decision process before the final value integration in the ventromedial PFC. However, although plausible, this interpretation is still speculative, because the present study does not provide direct evidence for the involvement of episodic future thinking in real-life self-control. Note that our results are compatible with the view that alternative forms of perspective taking, such as imagining the perspective of others, contribute to self-control. Future studies should combine EMA with more restricted experimental designs to clarify the roles of different forms of perspective taking in self-control.

To assess the ecological validity of multivariate brain patterns, we investigated associations between those patterns and daily self-control failures measured via EMA (Barrett & Barrett, 2001; Csikszentmihalyi & Larson, 1987; Stone & Shiffman, 1994). Compared with traditional paper-pencil self-reports EMA has strengths (e.g., it does not rely on memory, no need for aggregation, no artificial context) and weaknesses (measurement reactivity, participant burden, drop-out rate). Note that real-life self-control (EMA) and trait self-control (BSCS) were significantly correlated, but the size of this correlation is small. This is not surprising considering the different levels of analysis (trait self-control vs. situational self-control).

Note that multivariate brain patterns accounted for approximately 6% in variance in the EMA data. The fact that most of the variance in the EMA data remained unexplained is not surprising given the noisiness of the data. Our finding is important, because it illustrates how neuroimaging can add to the understanding of self-control.

A potential limitation of the task used in this study relates to the fact that participants were free to imagine for each item a realistic choice situation. Depending on the imagined situation, participants may make a decision consistent with short-term consequences that would not necessarily require self-control (e.g., drink alcohol at dinner) or consistent with long-term consequences that would likely require self-control (e.g., do not drink alcohol before driving). Thus, although the correlations of in-task decisions in line with participants’ ratings of long-term consequences with trait and real-life self-control suggest an involvement of self-control, we cannot be sure that participants experienced conflict or self-control during the task. Even if participants had not experienced self-control conflicts in our task, this would not invalidate the key finding that real-life self-control was associated with brain activity on trials, on which participants’ expressed preferences that were congruent with the subjective value of long-term (rather than the value of short-term) consequences of imagined behavioural options.

## Conclusions

This study revealed that real-life self-control is associated with multivariate brain patterns during preference decision making. The observed brain activations in the precuneus and in the angular gyrus are consistent with the hypothesis that self-control may be based on value modulation via the anticipation of future outcomes. Moreover, the results are consistent with the not mutually exclusive view that different forms of perspective taking, in particular imagining the perspective of others, contribute to self-control.

### Supplementary Information

ESM 1 (DOCX 100 kb)

## Data Availability

Group results of the fMRI analysis can be viewed and downloaded at neurovault (https://neurovault.org/collections/IYAIFAMK/). MVPA scripts can be downloaded at the TUD repository (https://cloudstore.zih.tu-dresden.de/index.php/s/i8SqDT2JHweGzsj).

## Ecological Momentary Assessment Questionnaire

**Table 1:** Ecological Momentary Assessment Questionnaire

Item	Wording	Response format	Condition for presenting item
1. *Desire*	Was there at some point during the last 60 minutes a situation where you had a desire and an opportunity to enact a certain behavior?	Yes/No	Alarm accepted
2. *Domain of desire*	Which of the following categories fits the desire most?^a^	19 options^a^	“Yes” response to Item 1
3. *Desire strength*	How strong was the desire on a scale from 1 (very weak) to 6 (very strong)?	Likert scale (1 to 6)	“Yes” response to Item 1
4. *Conflict*	In this situation, were you aware of any reason why you should not enact the desire?	Yes/No	“Yes” response to Item 1
5. *Conflict strength*	How strong was your conviction that you should not enact the desire on a scale from 1 (very weak) to 6 (very strong)?	Likert scale (1 to 6)	“Yes” response to Item 4
6. *Resistance*	Did you make an attempt to resist the desire?	Yes/No	“Yes” response to Item 4
7. *Enactment*	Did you (at least in part) enact the desire?	Yes/No	“Yes” response to Item 1

Original questionnaire was written in German.

^a^Eating; drinking (no alcohol); drinking (alcohol); smoking; taking other substance; using the Internet; playing a computer game; watching TV; buying something; gambling; exercising; sleeping; resting; retreating; misbehaving; socializing; having sex or intimacy; using the bathroom; other.

## Task Items

**Table 2:** ask Items

No.	Item (original)	Item (translated)
1	für eine Prüfung lernen	study for an exam
2	Hausaufgaben machen	do homework
3	Vokabeln lernen	learn vocabulary
4	eine Veranstaltung schwänzen	skipping an event
5	Pause überziehen	overrun a break
6	Drogen nehmen	take drugs
7	Alkohol trinken	drink alcohol
8	sich betrinken	get drunk
9	Joint rauchen	smoke a joint
10	Zigarette rauchen	smoke a cigarette
11	sich sonnen	sun bathe
12	Fastfood essen	eat fast food
13	schnell essen	eat fast
14	Süßigkeiten essen	eat sweets
15	viel essen	eat a lot
16	Geld sparen	save money
17	Geld ausgeben	spend money
18	sich einer Operation unterziehen	get surgery
19	sich impfen lassen	get vaccinated
20	Cola trinken	drink Coke
21	Fahrrad reparieren	repair bike
22	Fußboden wischen	clean the floor
23	Küche putzen	clean the kitchen
24	Toilette putzen	clean the toilet
25	Wäsche aufhängen	hanging up laundry
26	Wäsche waschen	make the laundry
27	Wohnung renovieren	renovate apartment
28	Wohnung saugen	vacuum the apartment
29	Lebensmittel einkaufen	buy groceries
30	laute Musik hören	listen to loud music
31	sich die Hände waschen	wash your hands
32	sich kalt duschen	take a cold shower
33	sich kratzen	scratch oneself
34	Affäre beenden	end affair
35	fremdgehen	cheat one somebody
36	schnell fahren	drive fast
37	früh aufstehen	get up early
38	früh ins Bett gehen	go to bed early
39	über jemanden lästern	gossip about someone
40	Computer spielen	play video games

## Details on the rating procedure

The full translation of the rating procedure is given below: “In the following questionnaire, we would like to know how you personally rate certain activities with regard to their long-term and short-term consequences. For example, one could imagine a person rating the short-term consequences of the item “repair bicycle” negatively (hands will get dirty, it costs time) and the long-term consequences positively (you don’t have to walk anymore). On the other hand, one could imagine a tinkerer who likes to repair his bicycle. The tinkerer maybe also rates the short-term consequences positively! Thus, it is important to rate the significance of the activities for you personally. It does not matter how other persons would rate the activity!”
