# Supplementary material for: Real-Life Self-Control is Predicted by Parietal Activity During Preference Decision Making: A Brain Decoding Analysis
Source: Cogn Affect Behav Neurosci. 2021 Jun 1;21(5):936–47. doi: 10.3758/s13415-021-00913-w (PMC8455489; doi:10.3758/s13415-021-00913-w)
Supplement: Supplementary file 1 — (DOCX 100 kb) [file 13415_2021_913_MOESM1_ESM.docx]

**Supplemental Material**

**Prediction of real-life self-control using k-fold cross-validation instead of leave-one-out cross-validation (LOOCV)**

An alternative to LOOCV, a procedure that could introduce prediction bias due to overfitting, is k-fold cross-validation. Here we report the results of a MVPA that is identical with the MVPA described in the main text except for the fact that LOOCA was replaced with k-fold cross validation, splitting the whole sample of 266 participants into 7 groups of 38 participants. Consistent with the results described in the main text, results of the MVPA based on 7-fold cross validation revealed that individual patterns of activity in bilateral angular gyrus and precuneus predict the probability of real-life self-control failures above chance (*r* = .159, *p* <.005, RMSE = .33, Figure S1).


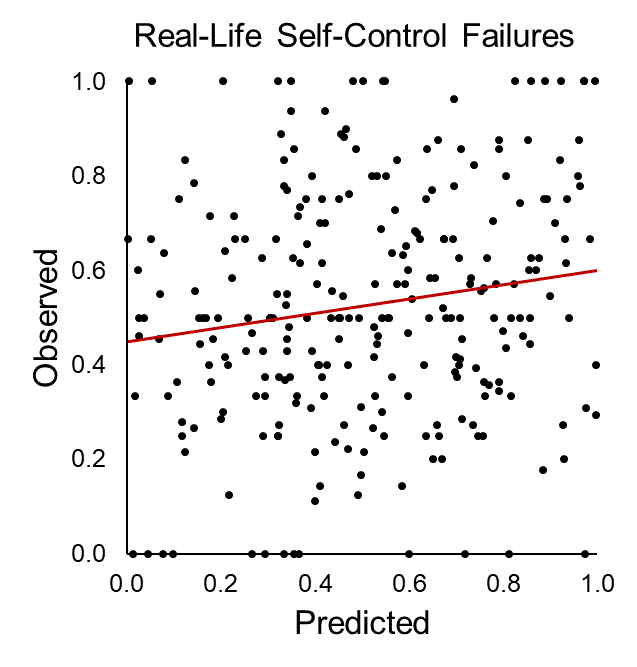


**Figure S1.** Prediction accuracy of an alternative MVPA based on 7-fold cross validation. Consistent with the result described in the main text, prediction of real-life self-control by MVPA based on 7-fold cross validation was correlated with observed self-control as measured by ecological momentary assessment (*r* = .159, *p* < .005, RMSE = .33).

**LOOCV for bilateral angular gyrus (left) and precuneus (right) separately.**

Note that real-life self-control was also predicted by a MVPA based on the bilateral angular cluster (*r* = .129, *p* < .018; RMSE = .31), but not by an MVPA based on the precuneus cluster alone (*r* = .031, *p* = .309; RMSE =.27; Figure S2).

**
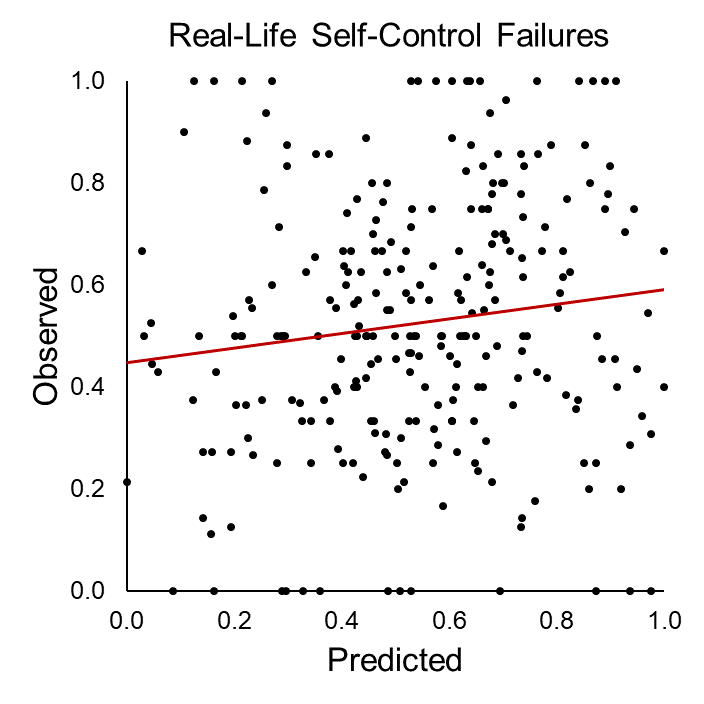

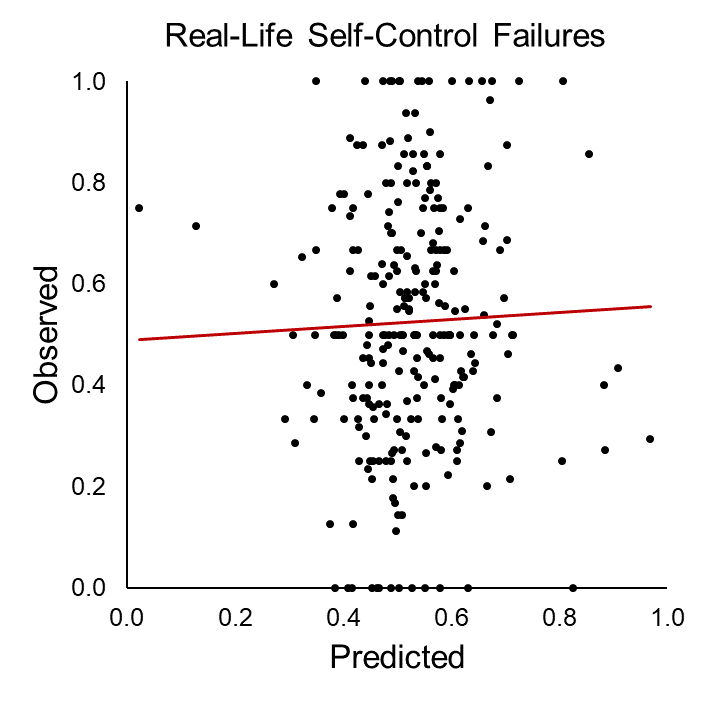
**

**Figure S2**. Prediction accuracy of two additional MVPA for bilateral angular gyrus (left) and precuneus (right) separately. Real-life self-control was predicted by a MVPA based on the bilateral angular cluster (*r* = .129, *p* < .018; RMSE = .31), but not by an MVPA based on the precuneus cluster alone (*r* = .031, *p* = .309; RMSE = .27).

**Prediction or real-life self-control based on univariate analyses**

In contrast to MVPA, univariate analyses revealed no association between real-life self-control and activity in bilateral angular gyrus and precuneus. This suggests that important additional information is encoded in the brain patterns, which is ignored in traditional univariate analysis. Furthermore, the usefulness of the univariate analysis approach is questioned by the limited range of predicted self-control failure probabilities based on univariate analysis (0.4-0.6).


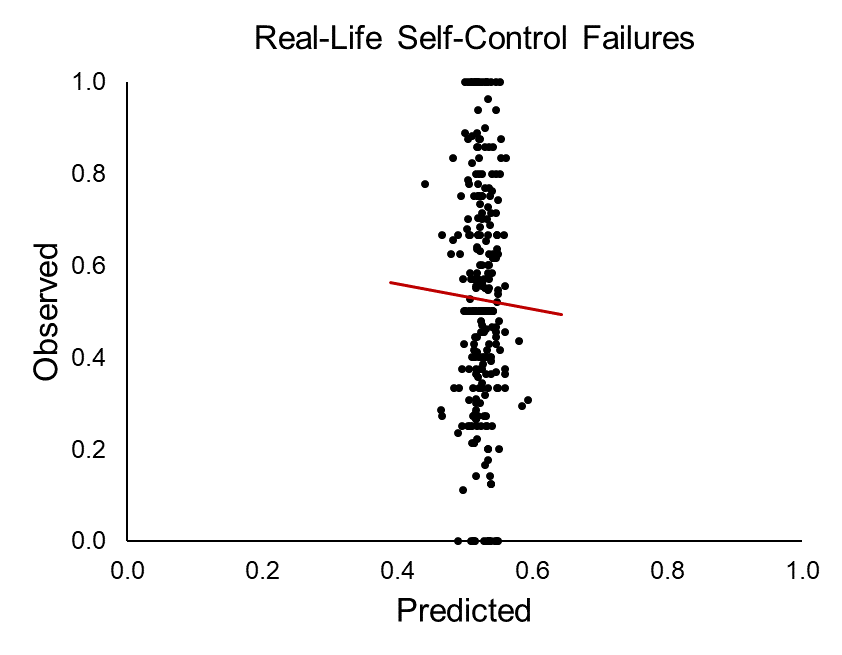


**Figure S3**. Prediction accuracy based on univariate analyses. Prediction of real-life self-control by univariate analyses was not correlated with observed self-control as measured by ecological momentary assessment (*r* = -.022, *p* = .637, RMSE = .25).
